# A novel orally active water-soluble inhibitor of human glutathione transferase exerts a potent and selective antitumor activity against human melanoma xenografts

**DOI:** 10.18632/oncotarget.2798

**Published:** 2015-02-14

**Authors:** Anastasia De Luca, Dante Rotili, Debora Carpanese, Alessia Lenoci, Laura Calderan, Manuel Scimeca, Antonello Mai, Elena Bonanno, Antonio Rosato, Cristina Geroni, Luigi Quintieri, Anna Maria Caccuri

**Affiliations:** ^1^ The NAST Centre for Nanoscience & Nanotechnology & Innovative Instrumentation, University of Tor Vergata, 00133 Rome, Italy; ^2^ Department of Drug Chemistry and Technologies, “Sapienza” University, 00185 Rome, Italy; ^3^ Department of Surgery, Oncology and Gastroenterology, University of Padova, 35128 Padova, Italy; ^4^ Department of Pharmaceutical and Pharmacological Sciences, University of Padova, 35131 Padova, Italy; ^5^ Department of Biomedicine and Prevention, University of Tor Vergata, 00133 Rome, Italy; ^6^ TMALab s.r.l., Spin-off of University of Tor Vergata, 00133 Rome, Italy; ^7^ Pasteur Institute, Cenci-Bolognetti Foundation, “Sapienza” University, 00185 Rome, Italy; ^8^ Istituto Oncologico Veneto IRCCS, 35128 Padova, Italy; ^9^ On-kòs Pharma Consulting, 20100 Milan, Italy; ^10^ Department of Experimental Medicine and Surgery, University of Tor Vergata, 00133 Rome, Italy

**Keywords:** Glutathione Transferase P1-1, c-Jun N-terminal Kinase, 6-((7-nitrobenzo[c][1,2,5]oxadiazoles, Human Melanoma Xenografts

## Abstract

We designed and synthesized two novel nitrobenzoxadiazole (NBD) analogues of the anticancer agent 6-((7-nitrobenzo[c][1,2,5]oxadiazol-4-yl)thio)hexan-1-ol (NBDHEX). The new compounds, namely MC3165 and MC3181, bear one and two oxygen atoms within the hydroxy-containing alkyl chain at the C4 position of the NBD scaffold, respectively. This insertion did not alter the chemical reactivity with reduced glutathione, while it conferred a remarkable increase in water solubility. MC3181 was more selective than NBDHEX towards the target protein, glutathione transferase P1-1, and highly effective *in vitro* against a panel of human melanoma cell lines, with IC_50_ in the submicromolar-low micromolar range. Interestingly, the cellular response to MC3181 was cell-type-specific; the compound triggered a JNK-dependent apoptosis in the BRAF-V600E-mutated A375 cells, while it induced morphological changes together with an increase in melanogenesis in BRAF wild-type SK23-MEL cells.

MC3181 exhibited a remarkable therapeutic activity against BRAF-V600E-mutant xenografts, both after intravenous and oral administration. Outstandingly, no treatment-related signs of toxicity were observed both in healthy and tumor-bearing mice after single and repeated administrations.

Taken together, these results indicate that MC3181 may represent a potential novel therapeutic opportunity for BRAF-mutated human melanoma, while being safe and water-soluble and thus overcoming all the critical aspects of NBDHEX *in vivo*.

## INTRODUCTION

Malignant melanoma is the most aggressive skin cancer, and its incidence continues to rise worldwide. In stage 0, I and II (localized) disease, surgical excision of the primary tumor is associated with a cure rate of over 95%. However, in stage III (regional lymph node metastasis) and stage IV (distant metastasis) disease, surgical interventions are of limited value, and systemic therapy becomes the only treatment option. Unlike dacarbazine, temozolomide and interleukin-2, the recently approved ipilimumab and vemurafenib drugs improve median overall survival in metastatic melanoma patients [1–3], but nonetheless there is still a high unmet need for more effective and safer systemic therapies.

Both intrinsic and acquired mechanisms are thought to be responsible for the multidrug-resistant phenotype of melanoma cells. Among these, overexpression of glutathione transferase (formerly glutathione *S*-transferase, GST) P1-1 may play an important role. This dimeric cytosolic protein is overexpressed in many untreated human tumors, including melanoma, and is well known to catalyze the detoxification of some electrophilic anticancer drugs via conjugation to reduced glutathione (GSH) [4–8]. Furthermore, GSTP1-1 is often co-expressed with the multidrug resistance-associated protein 1 (MRP1) in melanoma, and acts in a combined fashion with this export pump to protect melanoma cells from some cytotoxic agents [9, 10].

Beside its detoxifying function, GSTP1-1 has a regulatory role in signal transduction, interacting with different members of the Mitogen-Activate Protein Kinase (MAPK) pathway. Activation of c-Jun N-terminal kinase (JNK)-mediated MAPK signaling occurs in response to several anticancer agents including those causing DNA breaks, such as temozolomide [11]. The association of GSTP1-1 with both JNK1 and the up-stream adaptor protein TNF-Receptor Associated Factor 2 (TRAF2) results in impairment of the MAPK pathway, leading to inhibition of apoptosis [12–15].

This evidence prompted us to synthesize and screen non-GSH-peptidomimetic GSTP1-1 inhibitors bearing a nitrobenzoxadiazole (NBD) moiety. Among them, the most effective was NBDHEX. This compound behaves as a “mechanism-based inhibitor” of GSTP1-1 [16] leading to the activation of the apoptotic pathway in a variety of human cancer cell lines [17–20]. We recently demonstrated that NBDHEX exhibits *in vitro* cytotoxicity at low micromolar concentrations, and significant *in vivo* therapeutic activity towards Me501 and A375 human melanomas [21]. In addition, NBDHEX synergize with temozolomide both *in vitro* and *in vivo*, without worsening the myelotoxic activity of the methylating agent [22]. However, NBDHEX suffers from relatively low target selectivity being a high-affinity ligand of the widely-expressed GSTM2-2 [23], and from having poor water solubility, which limits its oral bioavailability and hinders the development of drug formulations suitable to parenteral administration. These findings led us to search for novel NBDHEX analogues with a better pharmacological profile.

Based on the crystal structure of NBDHEX bound to either GSTP1-1 or GSTM2-2 [24], we recently designed, synthesized and screened for water solubility, GSTP1-1 selectivity and *in vitro* tumor cell cytotoxicity, 40 new NBDHEX analogues [25]. As a further development of these studies, here we report the synthesis and chemical characterization of two additional NBDHEX analogues, namely MC3165 and MC3181; they are characterized by the presence of one and two oxygen atoms within the hydroxy-containing alkyl chain at the C4 position of the NBD scaffold, respectively. These compounds have been designed with the aim of increasing the water-solubility while minimizing the changes into the NBD nucleus, to avoid any significant drop of cytotoxic potency. Both compounds have been assayed for water solubility, chemical stability and ability to form a distinctive σ-complex intermediate with GSH [16]. Moreover, we examined their inhibition strength towards both GSTP1-1 and GSTM2-2, and the degree of selectivity towards GSTP1-1. The most promising compound, MC3181, was further studied *in vitro* both for ability to affect the interaction between GSTP1-1 and JNK1 or TRAF2, and for antitumor efficacy against a panel of human melanoma cell lines. Finally, host toxicity and antitumor efficacy of MC3181 upon its intravenous (i.v) or oral administration were assessed *in vivo* in healthy animals and mice bearing different human melanoma xenografts.

## RESULTS

### MC3165 and MC3181 preparation

**Figure 1:**
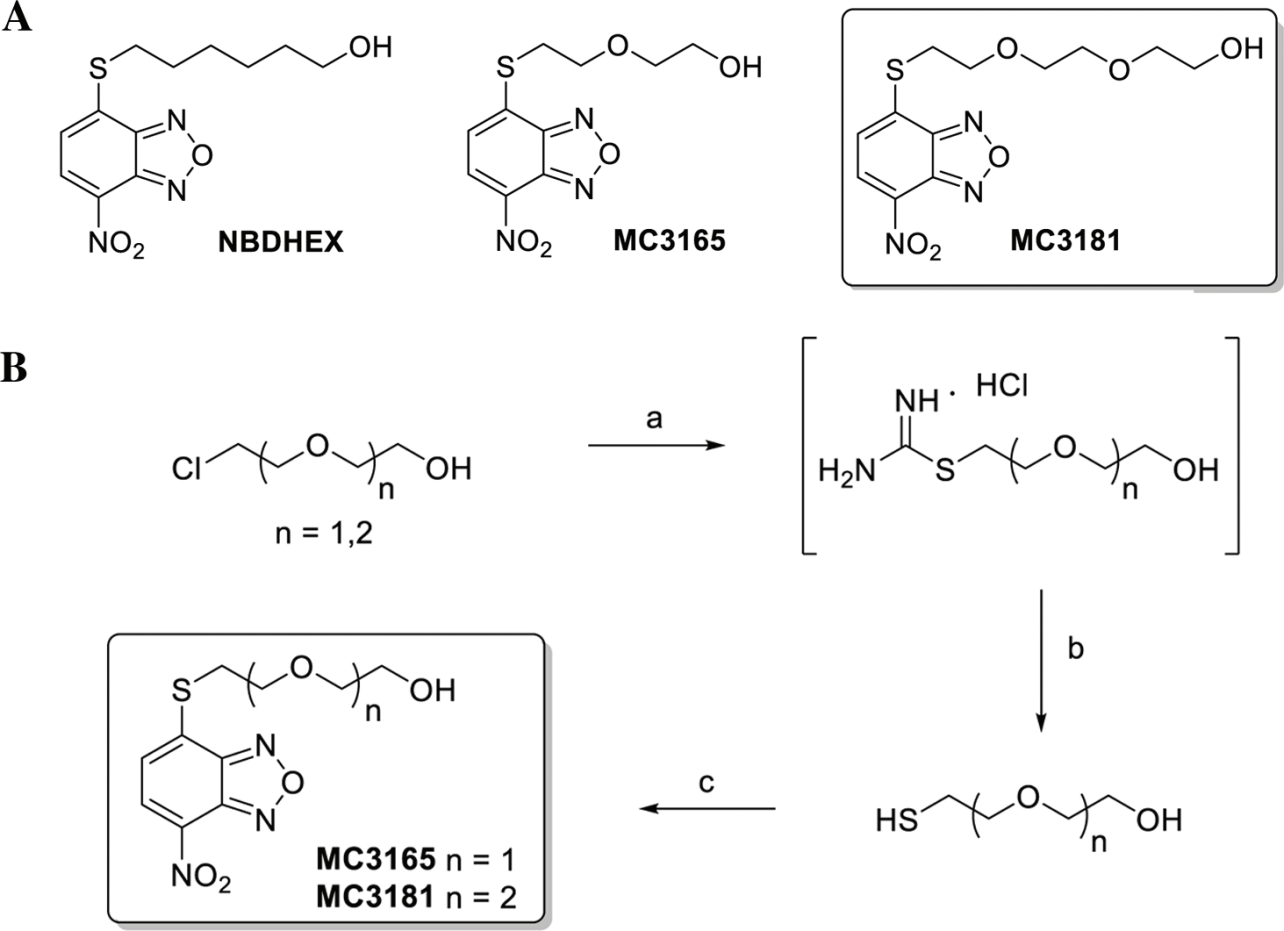
Structures and preparation of the NBDHEX derivatives MC3165 and MC3181 **(A)** Chemical structures of NBDHEX, MC3165 and MC3181. **(B)** Scheme of MC3165 and MC3181 preparation. The compounds were obtained by a nucleophilic displacement reaction between the commercial 4-chloro-7-nitrobenzo[*c*][1,2,5]oxadiazole and the 2-(2-mercaptoethoxy)ethanol and the 2-(2-(2-mercaptoethoxy)ethoxy)ethanol, respectively. Reagents and conditions: a) Thiourea, H_2_O, N_2_, reflux, 18h; b) NaOH 5N, N_2_, reflux, 3h; c) 4-chloro-7-nitrobenzo[*c*][1,2,5]oxadiazole, EtOH:H_2_O (0.3:1), pyridine, r.t., 4h.

As described in Fig. 1, MC3165 and MC3181 were obtained by a nucleophilic displacement reaction between the commercial 4-chloro-7-nitrobenzo[*c*] [1,2,5]oxadiazole and the 2-(2-mercaptoethoxy)ethanol and the 2-(2-(2-mercaptoethoxy)ethoxy)ethanol, respectively. The mercapto alcohols were prepared from the commercially available 2-(2-chloroethoxy)ethanol and 2-(2-(2-chloroethoxy)ethoxy)ethanol through a reaction with thiourea in refluxing water under an inert atmosphere, followed by the basic hydrolysis of the hydrochloride intermediates under reflux conditions. The 2-(2-mercaptoethoxy)ethanol and the 2-(2-(2-mercaptoethoxy)ethoxy)ethanol were finally reacted with the 4-chloro-7-nitrobenzo[*c*][1,2,5]oxadiazole in an ethanol:water (1:3) mixture, in the presence of pyridine at room temperature, providing the desired compounds, MC3165 and MC3181.

### Chemical and physical properties of MC3165 and MC3181

The molecular weights, extinction coefficients, and the aqueous solubility profiles at different pH values of MC3165, MC3181 and their parent compound, NBDHEX, are reported in Table 1.

**Table 1:** Molecular weights, extinction coefficients and solubility limits

Compound	Mol. Weight (Da)	ε 425 nm (mM^−1^cm^−1^)	Solubility (mM)		
			pH 7.4*	pH 5.0^	pH 2.0^
**NBDHEX**	297.3	15.0 ± 0.1°	0.1	0.2	0.2
**MC3165**	285.3	20.0 ± 0.4	2.0	1.9	2.0
**MC3181**	329.3	16.4 ± 0.2	5.0	8.0	9.0

* 0.1 M K-Phosphate buffer,

^ 0.1 M Na-Acetate Buffer, °433 nm.

Data represent means ± SD

Replacement of the *n*-hexanol chain of NBDHEX with a 2-(ethoxy)ethanol moiety (MC3165) led to a 20-fold increase in water solubility at all the examined pH values. Insertion of two oxygen atoms within the hydroxy-containing alkyl chain of NBDHEX (MC3181) further improved hydrophilicity resulting in a more than 50-fold increase in aqueous solubility.

### Spontaneous reactivity of MC3165 and MC3181 with GSH

**Figure 2:**
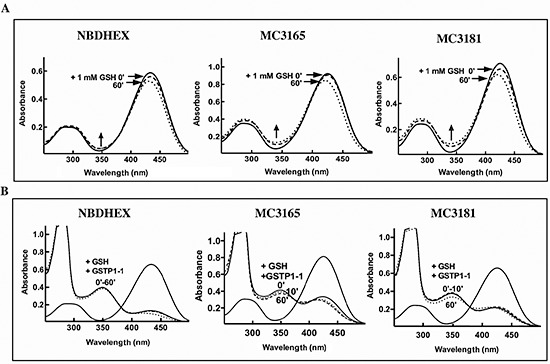
Reactivity with GSH of NBDHEX, MC3165 and MC3181 **(A)** UV-visible spectra of NBDHEX, MC3165 and MC3181 (40 μM, in 0.1 M potassium-phosphate buffer, pH 6.5, at 25°C) before (solid line), immediately after (dashed line), and 60 minutes after (dotted line) the addition of an excess of GSH (1 mM). Arrows show a small increase in the region around 350 nm indicative of the spontaneous formation of a σ-complex between the compound and GSH. **(B)** UV-visible spectra of NBDHEX, MC3165 and MC3181 (40 μM, in 0.1 M potassium-phosphate buffer, pH 6.5, at 25°C) before (solid line), 10 minutes (dashed line), and 60 minutes (dotted line) after addition of a stoichiometric amount (40 μM) of GSTP1-1 and of an excess of GSH (1 mM). Upon addition of GSTP1-1, formation of the typical σ-complex band centered at 350 nm was observed.

The potential of the novel NBD derivatives to spontaneously form GSH-conjugates, that might be rapidly extruded from the cell by specific membrane transporters such as MRP1/2 [26], was evaluated spectrophotometrically. Addition of an excess of GSH to MC3165, MC3181 or NBDHEX solutions in 0.1 M potassium phosphate buffer (pH 6.5) at 25°C, caused only a small decrease in the absorbance at 430 nm (less than 15% decrease of the absorbance value within 1 h), and a comparable increase in the absorbance band in the 350 nm region (Fig. 2A). Collectively these results indicate a negligible spontaneous reactivity of all of the examined compounds towards GSH.

### Sigma complex formation in the GSTP1-1 active Site

As already observed with NBDHEX [16], GSTP1-1 strongly increased the reaction rate between GSH and the new NBD derivatives. The spectral band of MC3165 and MC3181 in the 430 nm region disappeared immediately upon their incubation with GSH and GSTP1-1, and was replaced by a typical σ-complex band centered at 350 nm (Fig. 2B) [16]. The nature of the substituent bound to the sulphur atom influenced the stability of the σ-complex, which was in the rank order NBDHEX > MC3181 > MC3165 (Fig. 2B).

### Inhibition of GSTP1-1 and GSTM2-2 conjugation activity by MC3165 and MC3181

To evaluate the degree of selectivity of the novel NBD derivatives against the GSTP1-1 isoform, the IC_50_ values of each compound towards the catalytic activity of both GSTP1-1 and GSTM2-2 were determined, and used for calculation of a selectivity index (SI, IC_50GSTP1-1_/IC_50GSTM2-2_ ratio, see Table 2). While MC3165 had a SI comparable to that of its parent compound (Table 2), MC3181 displayed a 3-fold lower SI than NBDHEX, thus indicating a greater selectivity towards GSTP1-1. Based on these findings, MC3181 was selected for further *in vitro* and *in vivo* investigations as a potential therapeutic antimelanoma agent.

**Table 2:** Inhibitory activities of NBDHEX, MC3165 and MC3181 against GSTP1-1 and GSTM2-2

Compound	IC50 (μM)		Selectivity Index IC_50GSTP1-1_/IC_50GSTM2-2_
	GSTP1-1	GSTM2-2	
NBDHEX	0.8 ± 0.1	≤ 0.01	≥ 80
MC3165	4.0 ± 0.2	0.051±0.008	78
MC3181	2.6 ± 0.3	0.081±0.013	32

IC_50_ values were calculated by fitting the binding curves to the Equation 1

(see Methods section). Data represent means ± SD

### MC3181 disrupts protein-protein interactions involving GSTP1-1

**Figure 3:**
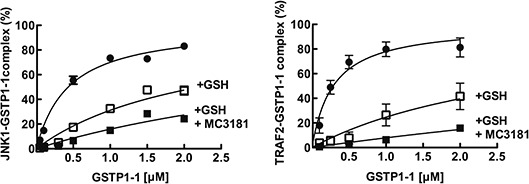
Effect of MC3181 on the Binding of GSTP1-1 to immobilized His-Tag JNK1α2 and TRAF2 **(A)** Immobilized His-Tag JNK1α2 (18 nM, left panel) or His-tagged TRAF2 C-terminal domain (5 nM, right panel) were incubated with increasing GSTP1-1 concentrations (ranging from 0.2 to 2 μM) (-●-). Alternatively, GSTP1-1 incubation was performed in the presence of either 1 mM GSH (-□-) or a mixture of 1 mM GSH and 8 μM MC3181 (-■-). The amount of GSTP1-1 bound was revealed by using an anti-GSTP1-1 specific antibody (ELISA). Data represent means ± SD of three independent experiments.

We recently demonstrated that NBDHEX is able to disrupt the interaction between GSTP1-1 and the MAPK pathway members JNK1 [14] and TRAF2 [15]. Therefore, the effects of MC3181 on these protein-protein complexes were evaluated. Under our experimental conditions, GSTP1-1 resulted bound to JNK1α2 and TRAF2 with K_d_ values in the nanomolar range (0.42 ± 0.02 μM and 0.28 ± 0.02 μM, respectively; Fig. 3 and Table 3). The presence of GSH remarkably affected the binding properties of GSTP1-1, shifting the K_d_ values for JNK1α2 and TRAF2 to 2.2 ± 0.1 μM and 3.0 ± 0.3 μM, respectively. Moreover, the addition of MC3181, further hindered the formation of the GSTP1-1-protein complexes leading to K_d_s > 5–10 μM.

**Table 3:** Protein-protein interaction between GSTP1-1 and JNK1α2 or TRAF2

Protein-protein complex	K_d_ (μM)			
	--	+ GSH	+ GSH+ NBDHEX	+ GSH+ MC3181
**JNK1α2-GSTP1-1**	0.42 ± 0.02	2.2 ± 0.1	> 10*	≥ 5
**TRAF2-GSTP1-1**	0.28 ± 0.02	3.0 ± 0.3	≥ 5^	≥ 10

The K_d_s of the complexes were calculated by fitting the binding curves to the Equation 1

(see Methods section). Data represent means ± SD. References * (14 ), ^ (15).

### *In vitro* antitumor efficacy of MC3181 towards a panel of human melanoma cell lines

The antitumor efficacy of MC3181 towards five cultured human melanoma cell lines was compared with that of NBDHEX. A375, G-361, MALME-3M and IST-MEL-1 are cell lines harboring the BRAF-V600E mutation, whereas SK23-MEL are BRAF wild-type melanoma cells. Results, summarized in Table 4, show that the IC_50_ values of MC3181 were in the low micromolar range (0.8–2.4 μM), and comparable to that recorded for NBDHEX

**Table 4:** Cell growth inhibition studies in human melanoma cell lines

Cell line	IC_50_ (μM)	
	NBDHEX	MC3181
A375	1.45 ± 0.14	2.31 ± 0.04
SK23-MEL	1.19 ± 0.09	1.79 ± 0.05
G-361	0.55 ± 0.03	0.78 ± 0.04
MALME-3M	1.80 ± 0.12	2.42 ± 0.16
IST-MEL-1	1.42 ± 0.05	0.92 ± 0.07

Cells were exposed to different concentrations (0.05–50 μM) of MC3181 or NBDHEX for 48 h. The IC_50_ values for each compound were then evaluated by the SRB assay

### Analysis of cell death induced by MC3181 in A375 and SK23-MEL cells

**Figure 4:**
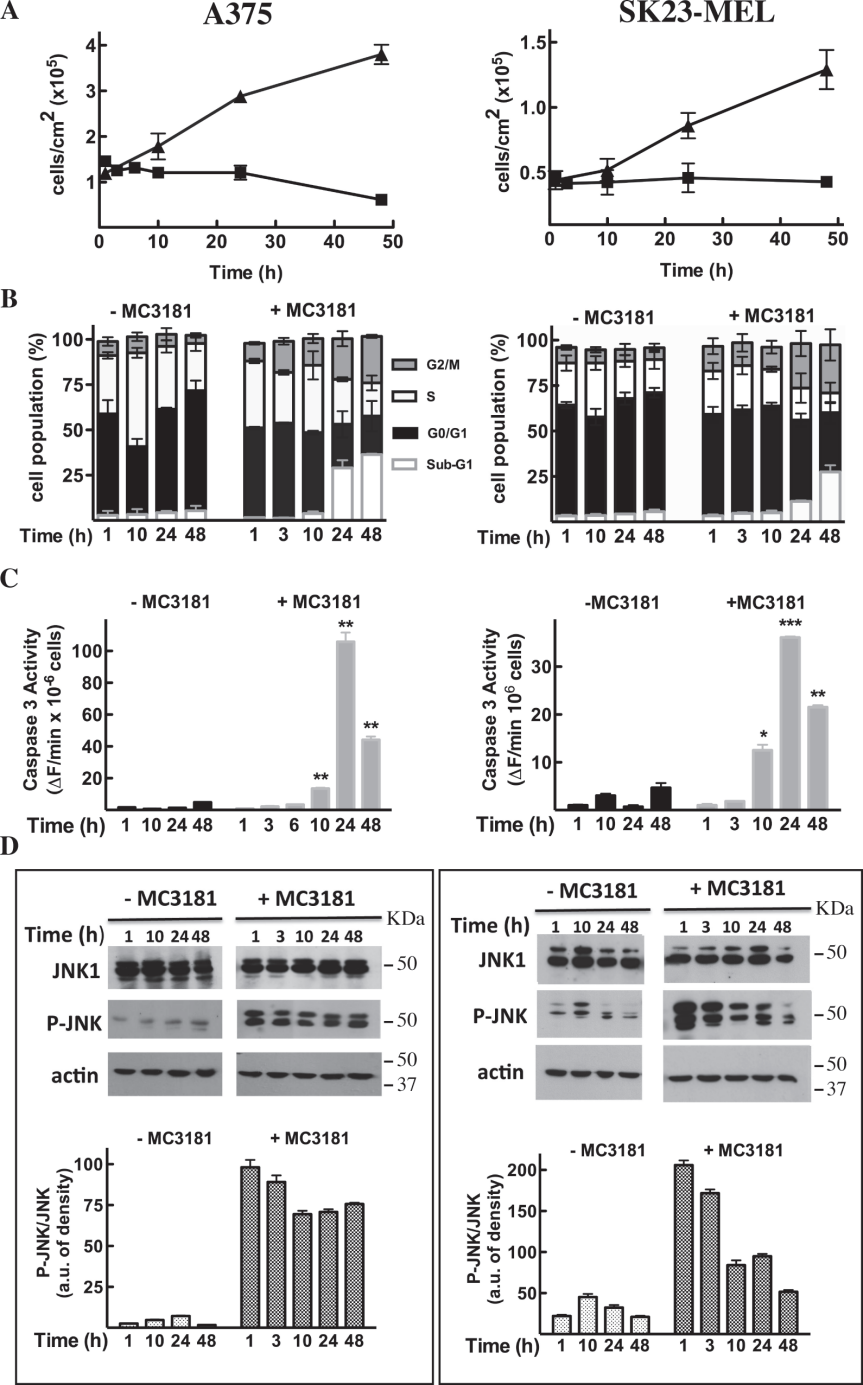
Effects of MC3181 on human melanoma cell lines A375 (left panels) and SK23-MEL (right panels) cells were treated with equitoxic concentrations of MC3181 (10 μM and 7 μM, respectively). **(A)** MC3181 treatment (-■-) induced a sustained growth inhibition in both A375 and SK23-MEL cell lines, while untreated cells (-▲-) were in active proliferation. **(B)** Cell cycle analysis of A375 and SK23-MEL cells exposed to MC3181 for 48 hrs. **(C)** Time-dependent caspase-3 activation in A375 and SK23-MEL cells treated with MC3181. **(D)** Time-dependent phospho-activation of JNK in A375 and SK23-MEL cells treated with MC3181; actin was used as loading control. Densitometric analysis revealed a sustained and prolonged activation of JNK in the A375 cell line, while phosphorylation of JNK was early and transient in SK23-MEL cells. Data represent means ± SD of four independent experiments; (*) *P* < 0.05, (**) *P* < 0.005 and (***) *P* < 0.0005.

A strong inhibition of cell proliferation was observed in both A375 and SK23-MEL cells treated with MC3181 at concentrations about 4-fold higher than their IC_50_ values, *i.e.* 10 and 7 μM, respectively (Fig. 4A).

**Figure 5:**
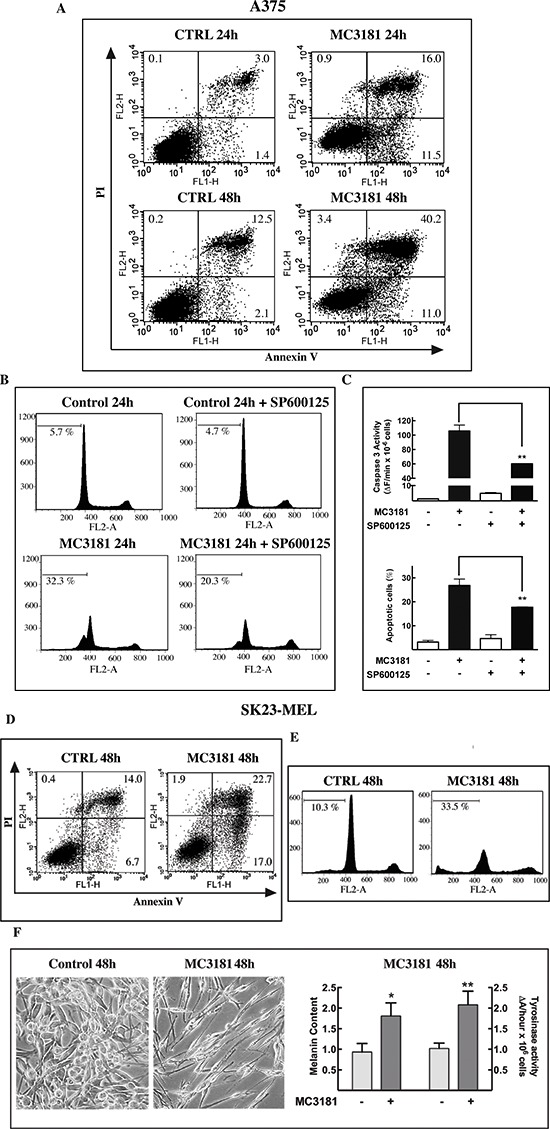
MC3181 triggers JNK-dependent apoptosis in A375 cells and morphological changes in SK23-MEL cells **(A)** The percentage of early apototic, late apoptotic, and necrotic A375 cells was evaluated by cytofluorimetric analysis of Annexin V versus PI staining, after 24 and 48 hrs incubation with 10 μM MC3181. **(B, C)** Pre-incubation of A375 cell line with the JNK inhibitor SP600125 (20 μM) significantly reduced the percentage of Sub-G1 (apoptotic) cells, and strongly suppressed caspase-3 activation. **(D)** Percentage of early apototic, late apoptotic, and necrotic cells and **(E)** of sub-G1 phase population in SK23-MEL cells following 48 hrs exposure to 7 μM MC3181. **(F)** Phase-contrast microscopy images (10X magnification, 3X digital magnification) show SK23-MEL cells with a typical triangular dendritic/spindle shape in control cultures, whilst bipolar spindle morphology is induced by 48 hrs incubation with MC3181 (7 μM). An increase of melanin content and tyrosinase activity were also observed in SK23-MEL cells after 48 hrs incubation with MC3181. Data represent means ± SD of three independent experiments.

Flow cytometry analysis of cell cycle perturbations induced by MC3181 in A375 cells revealed a time-dependent increase in the number of cells blocked in the G2/M phase and a concomitant increase in the amount of cells in the sub-G1 phase (about 27 and 36% increase after 24 and 48 hrs, respectively; Fig. 4B, left panel and Fig. 5, panels B and C). A noticeable cell cycle arrest in the G2/M phase was also observed in MC3181-treated SK23-MEL cells, whereas the drug-induced increase in the number of cells in sub-G1 phase was less pronounced than that recorded in A375 cells (about 10 and 20% increase after 24 and 48 hrs of treatment, respectively; Fig. 4B, right panel and Fig. 5, panel E). These differences translated into a different degree of caspase activation; a strong caspase-3 activity (Fig. 4C, left panel) was observed in drug-treated A375 cells, while MC3181 induced only a negligible increase of proteolitic activity in SK23-MEL cells (Fig. 4C, right panel).

To get better insight into the mechanism of cell death induced by MC3181, apoptosis was assessed by flow cytometry upon annexin V and propidium iodide (PI) staining. The analysis was performed after 24 and 48 hrs of exposure to the drug in the A375 cell line, and after 48 hrs of drug treatment in SK23-Mel cells, since, in these cells, apoptosis was negligible at 24 hrs. Results of these experiments are summarized in Figure 5. Early (Ann+/PI−) and late (Ann+/PI+) apoptotic cell death but not necrosis was recorded in both cell lines. The percentage of total apoptotic cells in A375 cells was approximately 23 and 37% at 24 and 48 hrs post-treatment, respectively (Fig. 5, panel A); the percentage of total apoptotic cells for SK23-Mel cells (Fig. 5, panel D) was about 20% after 48 hrs of treatment, in agreement with the results obtained with the PI staining.

### MC3181 activates JNK in A375 and SK23-MEL cell lines

Subsequent experiment examined the ability of MC3181 to activate the MAP kinase JNK1. Treatment of A375 cells caused a sustained increase in JNK phosphorylation, starting within 1 hour after the addition of the drug (Fig. 4D, left panel); a more transient phospho-activation of JNK was observed in SK23-MEL cells, treated with an equitoxic concentration of MC3181 (Fig. 4D, right panel).

Pretreatment of A375 cells with the JNK inhibitor SP600125 (20 μM), reduced by about 40% the increase in the number of cells in Sub-G1 phase (Fig. 5B and 5C), and by about 50% the increase of caspase-3 activity (Fig. 5C), elicited by a 24 hrs exposure to MC3181. Collectively, these data confirm the role of JNK in the apoptotic response triggered by MC3181 in A375 cells.

### MC3181 induces morphological changes in SK23-MEL cells

SK23-MEL cells are a mixture of spindle and polydendritic cells expressing markers indicative of cells at a late stage of melanocyte differentiation. A 48 hrs treatment with MC3181 caused clear morphological changes, shifting towards a bipolar spindle morphology (Fig. 5F). As the expression of late markers usually correlates with an increase of pigment content and a higher tyrosinase activity [27], we evaluated the impact of MC3181 treatment on cell tyrosinase activity and melanin content. Forty-eight hrs exposure to the drug, produced a significant increase in both melanin content (*p* < 0.05), and tyrosinase activity (*p* < 0.005), (Fig. 5C).

### Antitumor efficacy of MC3181 in human melanoma xenograft models

**Figure 6:**
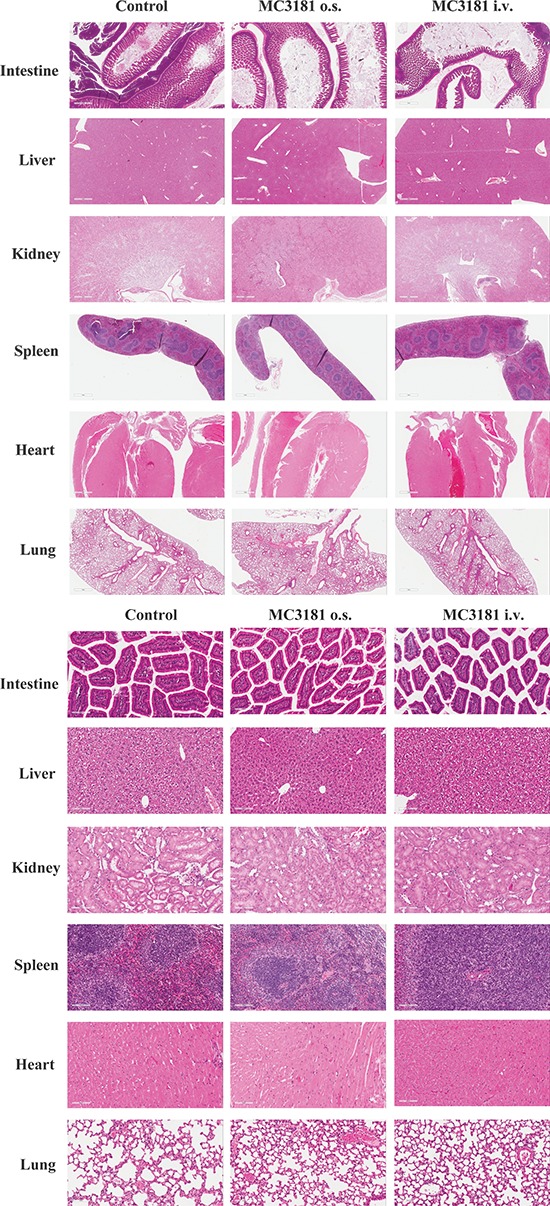
Histological analysis of tissue organs from CD-1 mice after 3 weeks of treatment with MC3181 Representative H&E stainings of intestine, liver, kidneys, spleen, heart, and lung sections; magnification, 2X (A) or 20X (B) Postmortem examination of organs/tissues did not show any microscopic finding

MC3181 doses and schedules were decided on the basis of both drug solubility properties and of explorative toxicology studies carried out in CD-1 mice, which were treated using either the i.v. or the oral route of administration. The maximum administrable dose of MC3181 (i.e. 8 mg/kg), which was dictated by its acqueous solubility, was given to mice using either the i.v. or the oral route and according to a q1dx5 schedule for 3 weeks without inducing apparent gross toxicity signs and weight loss (data not shown). Moreover, no microscopic differences were detectable in the structure of all organs between treated and untreated groups (Fig. 6A and 6B), thus demonstrating that the compound was safe at the tested dose schedule.

**Figure 7:**
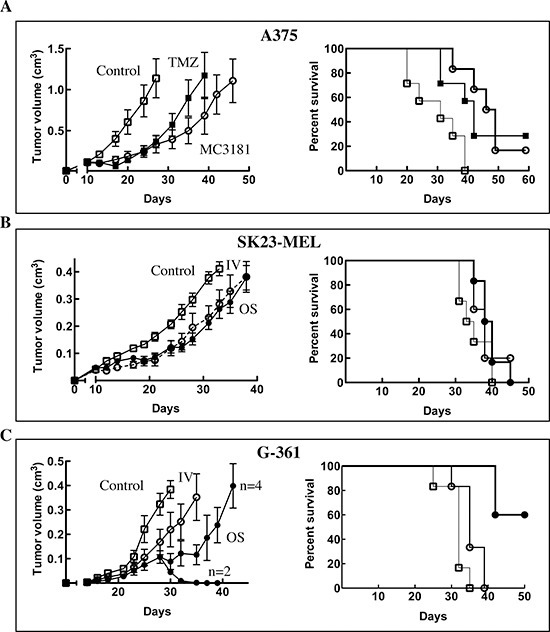
*In vivo* antitumor efficacy of MC3181 Mice were treated daily with 8 mg/kg of MC3181, administered intravenously (-○-) or orally (-●-) for three weeks. Alternatively, animals were treated i.p. with 100 mg/Kg of TMZ for 1 week (-■-). Control, mice were treated with drug vehicle (PBS) only (-☐-). Tumor growth (left panels) and Kaplan-Meier survival curves (right panels) of: **(A)** A375 fragment xenograft model (*n* = 7/group); **(B)** SK23-MEL and **(C)** G-361 cellular xenograft models (*n* = 6/group). In the left panels are reported the means ± SEM of tumor volumes determined in each group. Data shown for the trials in the A375 xenograft model are from a single experiment which was repeated twice with similar results; data shown for the trials in the SK23-MEL and G-631 xenograft models are representative of a single experiment.

Initially, the antitumor efficacy of MC3181 was investigated on the fast-growing A375 human melanoma model implanted s.c. as fragments, to better reproduce the tumor cell/stromal architecture. Animals (*n* = 7 mice/group) were treated i.v. with MC3181 (8mg/kg/d, q1dx5 for three weeks), or i.p. with a reference drug, TMZ (100 mg/kg/d, q1dx5), starting 10 days after tumor implant. MC3181 and TMZ treatments significantly delayed tumor growth from day 13 onward (*P* < 0.05; Fig. 7A, left panel); moreover, Kaplan–Meier analysis disclosed a significant increase in survival between treated and control mice (*p* < 0.05 for TMZ and *p* < 0.005 for MC3181, Fig. 7A, right panel), and fulfilled %ILS values of 53% and 35% for MC3181- and TMZ-treated animals, respectively.

A second set of experiments was performed in mice s.c.-injected with SK23-MEL or G-361 human melanoma cells (*n* = 6 mice/group), to compare the efficacy of MC3181 upon its administration by the i.v. or the oral route. In the case of the wild-type BRAF SK23-MEL cells, both treatments induced a certain degree of delay in tumor growth (Fig. 7B, left panel), but nonetheless effects appeared quite limited, and did not result in an improvement in survival (ILS < 20%) (Fig. 7B, right panel).

Conversely, treatment of the BRAF-mutated G-361 melanoma tumors produced highly remarkable data. Indeed, while both administration routes led to a significant delay in tumor growth, the oral delivery outperformed the i.v. counterpart, and was also capable of inducing stabilization and regression of the disease in half of the treated mice (Fig. 7C, left panel). These effects strongly impacted on the overall survival, as in the group receiving MC3181 orally, 50% of animals were still alive at the end of the observation period (Fig. 7C, right panel), thus clearly indicating the superior antitumor efficacy of the oral route.

**Figure 8:**
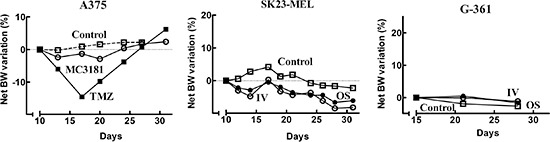
Net BW variation of mice bearing human tumor xenografts The percentage of net BW variation between the first day of treatment and the day of interest was evaluated according to Eq. 2. A375: control (-□-), TMZ i.p. (-■-), MC3181 i.v. (-○-); SK23-MEL and G-361: control (-□-), MC3181 i.v. (-○-), MC3181 oral (-●-). Overall, no treatment-related BW reduction or alterations in the animal behavior were observed in MC3181-treated mice, compared to control.

As in the case of *in vivo* toxicology trials, all of the animals evaluated in the therapeutic activity experiments were monitored for signs of drug toxicity by frequent observation and regular body weight (BW) measurements over the course of the study (Fig. 8). Overall, MC3181 was tolerated very well, with no signs of adverse effects and a decrease in BW lower than 3%. Moreover, macroscopic examination of the vital organs of treated animals did not reveal any alteration. The reference compound, TMZ, caused almost 15% of BW loss during the period of treatment, and a complete recovery only after its interruption (Fig. 8).

### Histological analysis

**Figure 9:**
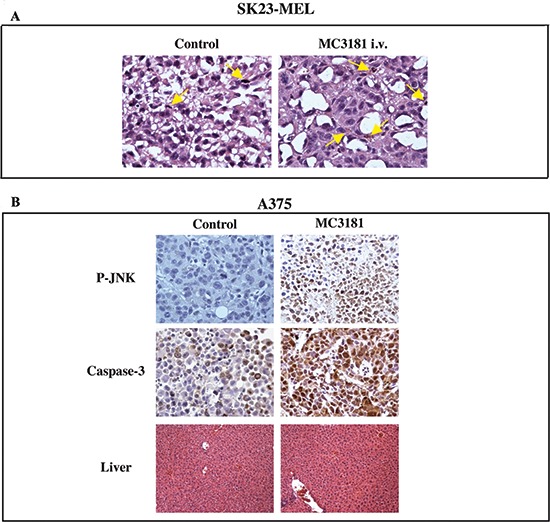
Immunohistochemical analysis **(A)** SK23-MEL tumor sample images: untreated xenografts were made of small to medium sized cells in active proliferation with organoid appearance (mitosis are indicated by arrows, left panel); MC3181 i.v.-treated xenografts, were composed of cells assuming epithelioid shape with large eosinophilic cytoplasm; in addition, some cells shifted towards melanin production (arrows, right panel). **(B)** P-JNK (6 hrs after treatment with MC3181) and caspase-3 stains (at the end of MC3181 treatment) (40X magnification) of A375 tumor xenografts. The drug caused a significant increase of phospho-JNK and caspase-3 stain, as compared to control tumors. Liver (lower panel) of both drug-treated and untreated animals did not show signs of toxicity.

At the end of the observation period, mice were sacrificed and an autopsy was performed. Gross examination of internal organs by a stereo-microscope did not show any significant lesion or metastatic nodules. Melanoma nodules were analyzed for morphology, proliferation and apoptosis. SK23-MEL control tumors showed small, highly proliferating cells (Fig. 9A, left panel). On the contrary, the MC3181-treated SK23-MEL tumors mainly contained epithelioid-shaped tumoral cells, characterized by large eosinophilic cytoplasm (Fig. 9A, right panel). In addition, some cells of drug-treated SK23-MEL xenografts showed melanin production, never observed in the control tumors.

In A375 tumors, we could not detect significant differences between the proliferation status of MC3181-treated and untreated tumors, as the extensive necrosis reduced the sampling areas to the tumor periphery. However, MC3181 treatment resulted in a significant increase of JNK phosphorylation and caspase-3 activity (see Fig. 9B). Moreover, higher apoptosis (about 20%) was still detectable in MC3181-treated tumor xenografts, compared to untreated controls (data not shown).

## DISCUSSION

The NBD derivative NBDHEX is a potent GST inhibitor, which has proven to be highly effective both *in vitro* and *in vivo* in various tumor models, including human melanoma xenografts [21, 22].

Recently, we designed, synthesized and characterized 40 new NBDHEX analogues, and found that the replacement of the hydroxyhexyl portion with an alkyl chain bearing polar substituents could be associated with a higher water solubility and a good cytotoxicity, compared to the parent compound [25]. Therefore, we extended our analysis to two novel alkyl analogues, named MC3165 and MC3181, which are characterized by the insertion of one and two oxygen atoms in the hexamethylene chain of NBDHEX, respectively.

Likewise NBDHEX, these new NBDs do not spontaneously react with GSH, whereas they form quite stable σ-complexes with this thiol in the GSTP1-1 active site. Overall, MC3181 showed overt improved chemical and physical properties compared to NBDHEX, i.e. a remarkable increase in water solubility and a higher selectivity towards the target protein, GSTP1-1. This prompted us to further investigate the mechanism of action of this novel compound, and to verify its activity at both cellular and *in vivo* levels.

MC3181 proved to be highly cytotoxic towards a panel of different human melanoma cell lines, as indicated by its IC_50_ values that were comparable, or in some cases slightly superior, to those recorded for the parent drug, NBDHEX. In particular, MC3181 efficiently inhibited proliferation of both the amelanotic and BRAF-mutated A375 cells, and of the melanotic and BRAF wild-type SK23-MEL cell line. On the other hand, the drug disclosed a different ability in inducing apoptosis in these two cell lines, suggesting a propensity for MC3181 to exert its antitumor activity against BRAF-mutated melanoma cells. Indeed, such observation was confirmed *in vivo*, as MC3181 proved not to be very effective in reducing tumor volume in SK23-MEL xenografts. However, unlike the untreated ones, MC3181-treated tumors, were characterized by cells with a large eosinophilic cytoplasm and producing melanin, consistent with the increased melanogenesis observed *in vitro*.

Conversely, in the A375 model, both TMZ and MC3181 showed a comparable efficacy, but with remarkable benefits in terms of toxicity profile for MC3181.

Notably, administration of MC3181 in G-361 tumor-bearing mice revealed a correlation between its antitumor activity and the route of administration, as the oral treatment led not only to a powerful tumor growth inhibition, but also produced a significant improvement in the overall survival. Again, it has to be stressed that the treatment-related signs of toxicity were negligible or completely absent.

With regard to the mechanism of action, MC3181 appeared to disrupt the interaction of GSTP1-1 with both JNK and the scaffold protein TRAF2, which is essential for JNK activation. Indeed, multiple cellular pathways are implicated in the development of melanoma and drug resistance, and therefore their inhibition is considered to be a valid therapeutic strategy for melanoma patients. In particular, the MAP kinase MEK is constitutively activated in the BRAF mutated cells, and it is a target for inhibitors [28]. However, melanoma can be also targeted with selected kinase activators; in fact, several anticancer agents require the activation of the MAP kinase pathway mediated by JNK and p38 [29]; accordingly, our evidences confirm that the proapoptotic activity of MC3181 is dependent on JNK activation.

Another important consequence of our findings is the possibility to exploit the levels of activated JNK (phospho-JNK) as a biomarker of drug responsiveness to be utilized for patient selection, and to monitor tumor response.

Collectively, these results indicate that MC3181 is an agent endowed with a high safety profile and a good water-solubility, thus overcoming all of the critical aspects of NBDHEX administration *in vivo*. As this compound resulted active upon oral administration, and proved to be therapeutically effective against BRAF-mutated tumors at non-toxic doses, it may represent a high compliant new therapeutic opportunity for the treatment of melanoma patients.

## METHODS

### Recombinant proteins and chemicals

Human GSTM2-2, GSTP1-1, JNK1a2 and TRAF2 were expressed in *Escherichia coli* and purified as previously described [14, 15, 30]; protein concentration was determined using the Lowry colorimetric assay [31]. Unless specified otherwise, all chemicals were purchased from Sigma-Aldrich S.r.l. (Milan, Italy).

### Chemistry

Melting points were determined on a Buchi 530 melting point apparatus. ^1^H-NMR spectra were recorded at 400 MHz using a Bruker AC 400 spectrometer; chemical shifts are reported in δ (ppm) units relative to the internal reference tetramethylsilane (Me4Si). Mass spectra were recorded on a API-TOF Mariner by Perspective Biosystem (Stratford, Texas, USA); samples were injected by an Harvard pump using a flow rate of 5–10 μL/min, and infused in the Electrospray system. All compounds were routinely checked by TLC and ^1^H-NMR. TLC was performed on aluminum-backed silica gel plates (Merck DC, Alufolien Kieselgel 60 F254) with spots visualized by UV light or using a KMnO_4_ alkaline solution. All solvents were reagent grade and, when necessary, were purified and dried by standard methods. Concentration of solutions after reactions and extractions involved the use of a rotary evaporator operating at reduced pressure of ~ 20 Torr. Organic solutions were dried over anhydrous sodium sulfate. All chemicals were purchased from Sigma-Aldrich s.r.l., Milan (Italy) or from TCI Europe N.V., Zwijndrecht (Belgium), and were of the highest purity. As a rule, samples prepared for physical and biological studies were dried in high vacuum over P2O5 for 20h at temperatures ranging from 25 to 40°C, depending on the sample melting point.

#### General procedure for the preparation of 2-(2-((7-nitrobenzo[*c*] [1,2,5]oxadiazol-4-yl)thio)ethoxy)ethan-1-ol (MC3165) and 2-(2-(2-((7-nitrobenzo[*c*][1,2,5]oxadiazol-4-yl)thio)ethoxy)ethoxy)ethanol (MC3181)

**Example: 2-(2-(2-((7-nitrobenzo[*c*][1,2,5]oxadiazol-4-yl)thio)ethoxy)ethoxy)ethanol (MC3181).**

To a solution of 2-(2-(2-chloroethoxy)ethoxy)ethanol (1.74 mL, 12 mmol ) in 20 mL of H_2_O, thiourea is added (1.37 g, 18 mmol) and the resulting mixture is stirred under nitrogen atmosphere for 30 minutes; then, the reaction is refluxed for 18 hrs, always under an inert atmosphere. The mixture was then cooled at room temperature, a 5N NaOH solution (15 mL) added and the reaction refluxed again for 3 hrs. The solution obtained was acidified at 0–5°C with concentrated HCl until pH = 4 and the aqueous phase was extracted with CHCl_3_ (4 × 40 mL). The combined organic phases were therefore dried over anhydrous Na_2_SO_4_, filtered and evaporated under reduced pressure to provide 1.68 g of 2-(2-(2-mercaptoethoxy)ethoxy)ethanol as a colorless oil that was used in the next step without further purification. ^1^H-NMR (CDCl_3_): δ 1.60 (t, 1H, CH_2_O*H*), 2.45 (s (*broad)*, 1H, *H*SCH_2_), 2.70 (q, 2H, CH_2_C*H_2_*SH), 3.67 (m, 8H, CH_2_C*H_2_*OC*H_2_*C*H_2_*OC*H_2_*CH_2_), 3.73 (t, 2H, SCH_2_C*H_2_*OH).

To a solution of 4-chloro-7-nitrobenzo[*c*][1,2,5]oxadiazole (575 mg, 2.89 mmol) in 6 mL of a mixture EtOH:H_2_O (0.3:1) were added in sequence pyridine (0.54 mL, 6.8 mmol) and 2-(2-(2-mercaptoethoxy)ethoxy)ethanol (417.2 mg, 2.51 mmol). The mixture was stirred at room temperature for 4 hrs. At the end of the reaction the aqueous mixture was extracted with CHCl_3_ (4 × 10 mL). The combined organic phases were then dried with anhydrous Na_2_SO_4_, filtered and evaporated under reduced pressure to obtain a brown solid crude, which was purified by column chromatography on silica gel eluting with a mixture CHCl_3_/CH_3_OH (150:1) to give the product as a yellow solid. Mp: 38–40°C (χ *n*-Hexane). Yield: 46%. ^1^H-NMR (DMSO): δ 3.38-3.42 (t, 2H, C*H_2_*OH), 3.45-3.60 (m, 8H, CH_2_C*H_2_*OC*H_2_*C*H_2_*OC*H_2_*CH_2_), 3.83 (t, 2H, CH_2_C*H_2_*S), 4.56 (t, 1H, CH_2_O*H*), 7.58 (d, 1H, *H* benzoxadiazole ring), 8.58 (d, 1H, *H* benzoxadiazole ring). MS-ESI m/z: 330 [M+H]^+^.

### Solubility limits in aqueous buffers

Different amounts of MC3181 and MC3165 were dissolved in a fixed volume of 0.1M potassium phosphate buffer pH 7.4, or 0.1 M sodium acetate buffer at pH 5.0 or 2.0. The solutions obtained were separated from any undissolved matter by centrifugation at 15,000 g and evaluated for drug concentration by recording compound absorbance at 425 nm, using molar extinction coefficients calculated at the same wavelength with diluted standard solutions, prepared in the same matrices.

### Reactivity towards GSH

The spontaneous reactivity of the NBDs with GSH was determined at 25°C by recording the UV-visible spectrum of each compound (40 μM) incubated with 1 mM GSH, in 0.1 M potassium phosphate buffer, pH 6.5, at different time points.

The ability of GSTP1-1 to stabilize the intermediate σ-complex formed between GSH and the compounds, was evaluated by recording the spectrum of each molecule (40 μM), in 0.1 M potassium phosphate buffer, pH 6.5, before and after the addition of a stoichiometric amount of GSTP1-1 and 1 mM GSH.

### Inhibition of GSTP1-1 and GSTM2-2 conjugation activity

GST activity was determined spectrophotometrically at 340 nm (ε = 9,600 M^−1^ cm^−1^), as previously reported [32]. Inhibition experiments were performed by measuring GSTP1-1 (20 nM) or GSTM2-2 (5 nM) activities in the presence of various amounts of the selected NBD derivative (from 0.1 to 20 μM with GSTP1-1, and from 0.005 and 1 μM with GSTM2-2). Fifty percent inhibitory concentration (IC_50_) values were calculated by fitting the data to Equation 1, where ν is the percentage of the saturated binding sites; [P]_t_ is the total GST concentration and [L]_t_ is the total inhibitor concentration, assuming a 1:1 interaction stoichiometry between the inhibitor and one GST subunit, as well as that the binding sites are equal and independent.

$$v=100\frac{{\left[P\right]}_{t}+{\left[L\right]}_{t}+IC_{50}-\sqrt{{\left({\left[P\right]}_{t}+{\left[L\right]}_{t}+IC_{50}\right)}^{2}-4{\left[P\right]}_{t}{\left[L\right]}_{t}}}{2{\left[P\right]}_{t}}$$ (Eq. 1)

### Protein-protein interaction

The protein-protein interaction between GSTP1-1 and JNK1α2 or TRAF2 recombinant proteins was evaluated in the absence or in the presence of either 1 mM GSH or a mixture of 1 mM GSH and 8 μM MC3181, as previously described [14, 15]. The equilibrium dissociation constants (K_d_) of the complexes were calculated by fitting the binding curves to the Equation 1, where ν is the percentage of the saturated binding sites, [P]_t_ is the total concentrations of TRAF2 or JNK1α2 and [L]_t_ is the total concentrations of GSTP1-1 (monomers).

### Cell culture and cell treatments

The following human melanoma cell lines were used throughout the study: A375, MALME-3M (American Type Culture Collection, ATCC; Manassas, VA); G-361, IST-MEL-1 (Interlab Cell Line Collection, ICLC, Genova, Italy); SK23-MEL cells were originally provided by Dr. T. Boon (Ludwig Institute for Cancer Research, Belgium).

All cell lines were profiled using DNA fingerprinting technology (AmpFlSTR Identifiler Plus PCR Amplification kit, Applied Biosystems) according to the manufacturer's protocol. The Short Tandem Repeat (STR) profiles of the analyzed cell lines were compared to DNA fingerprinting databases (i.e., ATCC, DMSZ). All the profiles analyzed showed a similarity higher than 80%.

A375 and SK23-MEL cells were grown in Dulbecco's MEM (EuroClone, Milan, Italy). IST-MEL-1 and MALME-3M were maintained in RPMI 1640 medium (EuroClone). G-361 cells were maintained in Mc Coy's medium (EuroClone). All culture media were supplemented with 10% (v/v) FBS, 2 mM L-glutamine, 100 U/ml penicillin, and 100 mg/ml streptomycin (Lonza, Basel, Switzerland). Growth medium for IST-MEL-1, G-361 and SK23-MEL, cells was also supplemented with 25 mM HEPES (Lonza); medium for IST-MEL-1, MALME-3M and G-361 was also enriched with 1 mM sodium pyruvate (Lonza). Cultures were maintained at 37°C in a humidified atmosphere containing 5% CO_2_.

For *in vitro* antitumor efficacy studies, cells (2×10^4^ cells/cm^2^) were seeded in 96-well plates and cultured for 24 h, after which they were exposed to different concentrations (0.05–50 μM) of MC3181 or NBDHEX for 48 h. The IC_50_ values for each compound were then evaluated by the SRB assay [33].

The proapoptotic and antiproliferative effects of MC3181 were evaluated in A375 and SK23-MEL cell lines seeded in 75 cm^2^ flask (Corning) at a density of 20,000 cells/cm^2^. Forty-eight hrs after plating, cells were exposed to equitoxic concentrations of MC3181 (10 and 7 μM for A375 and SK23-MEL, respectively), then were harvested at different time points, counted using a Neubauer Chamber (after 1:1 dilution in Trypan Blue), and subjected to further analysis.

In a separate set of experiments, the A375 cell line was exposed to the JNK inhibitor SP600125 (20 μM), which was added 1 h before the addition of MC3181.

### Western blot analysis

The cell pellet obtained at each time point was lysed as previously reported [17] and the protein concentration was determined using the Lowry colorimetric assay. Proteins (40 μg) were loaded on 12% SDS-polyacrylamide gel and transferred onto a PVDF membrane (GE Healthcare, Chalfont St. Giles, UK). Anti-P-JNK (Thr183 Tyr185) (Cell Signaling, Beverly, MA, USA), anti-JNK (Cell Signaling) and anti-β actin were used as primary antibodies. Anti-rabbit or anti-mouse secondary antibodies (Cell Signaling) were revealed with the ECL LiteAblot Extend (EuroClone). The ImageJ software was used to analyze the band intensities.

### Cytofluorimetric analysis

Cell cycle analysis was performed at different times after treatment with equitoxic concentrations of MC3181. A375 and SK23-MEL cells, were fixed with 70% ethanol overnight, stained with PI staining buffer (50 mg/ml PI, 10 mg/ml RNAsi and 1% Triton X-100) and analyzed by a FACSCalibur (BD Bioscence, San Jose, CA, USA). The percentage of early apoptotic, late apoptotic, and necrotic cells was determined by simultaneous staining of cells with PI (2 μg/ml) and Annexin V-FITC (0.5 μg/ml) (Sigma Aldrich), according to manufacturer's protocol. Flow cytometric data were analyzed by the FlowJo 8.8.6 software (Tree Stare, Inc, Ashland, OR, USA).

### Assessment of caspase-3 activity

Apoptosis was confirmed by measuring caspase-3 activity, at 25°C, in total cell lysates, using the model fluorescent peptide substrate *N*-Acetyl-Asp-Glu-Val-Asp-7-amido-4-trifluoromethylcoumarin (Ac-DEVD-AFC). Proteolytic cleavage of Ac-DEVD-AFC resulted in a fluorescence emission at 505 nm (excitation at 400 nm) [17]. Caspase activity was expressed as change in fluorescence per min, per 10^6^ cells.

### Determination of SK23-MEL melanin content and tyrosinase activity

Melanin content and tyrosinase activity of SK23-MEL cells were determined after 48 hrs incubation with MC3181 (7 μM). Cells (1×10^6^) were lysed in 1M NaOH at 100°C, for 1 hour and then centrifuged at 15,000 g, for 20 minutes. The melanin content of the resulting supernatant was evaluated spectrophotometrically at 490 nm, to avoid interference from MC3181. Tyrosinase activity was examined by measuring spectrophotometrically at 475 nm the rate of L-DOPA oxidation to dopachrome over 1 hour at 37°C. Cells [1×10^6^ in 40 μl of 20 mM potassium phosphate buffer (pH 6.8) containing 1% Triton X-100 and 1 mM PMSF] were disrupted by freezing and thawing and centrifuged at 15,000 g for 20 minutes at 4°C. The assay was started by addition of 100 μl of 2 mg/ml L-DOPA to 40 μl of supernatant. Tyrosinase activity was expressed as change in absorbance, per hour, per 10^6^ cells.

### *In vivo* explorative toxicology studies in mice

Procedures involving animals and their care were in conformity with institutional guidelines that comply with national and international laws and policies (D.L. 116/92 and subsequent implementing circulars), and experimental protocols were approved by the local Ethical Committee of Padua University (CEASA).

Multiple-dose toxicology studies were carried out in seven-week-old female CD-1 mice (Charles River, Calco, Italy). Animals were randomly assigned to each experimental group (*n* = 3/group) and were treated orally or intravenously with MC3181 (8 mg/kg dissolved in PBS), or PBS only (control group) for 5 consecutive days (q1dx5) followed by a 2-day wash-out. After 3 weeks of treatment, mice were sacrificed and organs collected for tissue injury assessment by H&E staining. Briefly, organs (intestine, liver, kidneys, spleen, heart, and lungs) were formalin-fixed (PFA 1%, Thermo Scientific, Milan, Italy) for 2 hrs and then transferred in PBS 20% sucrose (Sigma, Milan, Italy) for the H&E staining.

Toxicity was also evaluated in tumor-bearing mice on the basis of macroscopic autopsy findings, mainly in terms of alteration to the size and color of vital organs, and net BW reduction. The percentage of net BW variation, between the first day of treatment and sacrifice day (or day of interest), was evaluated according to the following equation:

$$\text{\% net BW variation }=\frac{\text{net BW at observation day }-\text{net BW at first day of treatment}}{\text{net BW at first day of treatment }}\times 1\text{00}$$ (Eq. 2)

### *In vivo* human melanoma models and treatment

All *in vivo* experiments were performed using 6- to 8-wk-old male or female immunodeficient mice of different strains purchased from Charles River Laboratories (Calco) and housed in specific pathogen-free animal facilities.

With regard to the *in vivo* fragment tumor model, A375 cells were injected (1×10^7^) subcutaneously (s.c.) into the right flank of an athymic Nude-Foxn1nu female mouse, and the established primary tumor was then removed and cut into fragments (2–3 mm diameter) and implanted s.c. into recipient mice. Animals were randomly divided in groups of 7 mice and treatment was initiated when a tumor volume of 0.12 ± 0.01 cm^3^ (mean ± SEM) was reached.

SK23-MEL and G-361 cells were injected s.c. (in both cases, 1×10^6^) into the right flank of NOD/SCID and SCID male mice, respectively. Animals were randomly divided in groups of 6 mice and treatments were started at day 10 (SK23-MEL) and 14 (G-361), when tumors were clearly palpable.

MC3181, dissolved in PBS, was administered i.v. or orally q1dx5 for three weeks at a dose of 8 mg/kg/day.

The reference drug temozolomide, dissolved in DMSO/PBS 1:1 to a final concentration of 10 mg/ml, was administered i.p. q1dx5 at a dose of 100mg/kg/day.

Control groups consisted of PBS-administered mice.

### Evaluation of antitumor efficacy *in vivo*

Primary tumor growth was determined for all animals by caliper measurement (minor and major axis length), three times per week, until they were sacrificed. Primary tumor volume was determined using the following equation:

$${\text{V (cm}}^{\text{3}})=\frac{(\alpha^{\text{2}}\times \beta \text{)}}{2}$$ (Eq. 3)

where α and β are the minor and major axes, respectively.

The efficacy of the treatment was also analyzed by calculation of the percentage of Increase in Life Span (% ILS), since this analysis gives an evidence of clinical potency [34]. Survival endpoint for each animal was fixed at the day when tumor volume reached 1.0 cm^3^ (A375) or 0.4 cm^3^ (SK23-MEL and G-361), and data were used to plot the relative Kaplan-Meier curves. Treatment efficacy was then evaluated by comparing the Median Survival Time (MST) of treated group with the MST of control group, and expressed as percentage of ILS:

$$\%\text{ ILS}=[100\times \frac{{(\text{MST}}_{\text{treated group}})}{{(\text{MST}}_{\text{control group}})}]-100$$

(Eq. 4)

Treatment was considered effective when % ILS > 20%.

### Immunohistochemical analysis

At sacrifice, tumors were removed, formalin-fixed and paraffin embedded. All internal organs were fixed and paraffin embedded for the morphological study to evaluate drug effects in normal tissues. Three μm-thick sections were cut and stained with haematoxylin and eosin (H&E) [35]. Two observers blindly classified the tissues for necrotic areas, evaluated as percent of total area, mitosis number in 10 high power field randomly selected away from the ischemic necrosis.

Tissue-xenografts treated with 8 mg/kg of MC3181 were studied by immonohistochemistry to identify the activation of JNK (after 6 hrs) and apoptosis, characterized by immune-staining for Caspase3 (at the end of treatment). Briefly, 3-μm-thick sections were pre-treated with EDTA citrate pH 7.8, for 30 min, at 95°C and then incubated respectively with rabbit monoclonal anti-Phospho-SAPK/JNK (1:100, 81E11, Cell Signaling, Danvers Massachusetts, USA) and rabbit polyclonal anti-Caspase3, for 60 min (1:100, PA5-16335, ThermoFisher Scientific, Walthman, MA, USA). Washing were performed with PBS 4% + Tween20, pH 7.6, by UCS diagnostic. Reactions were revealed by HRP – DAB Detection Kit (UCS diagnostic).

### Statistical analysis

All the *in vitro* experiments were repeated at least three times; results are presented as means ± SD. Statistical evaluation was done using the Student's *t*-test (two tailed).

Tumor volumes in *in vivo* studies (presented as means ± SEM) were analyzed by one-way analysis of variance (ANOVA) followed by Bonferroni test for multiple comparisons. Kaplan-Meier product-limit method was performed to estimate the survival curves, and comparison of survivals between groups was performed using the log-rank test. The results were considered to be statistically significant at *P* < 0.05.

## Abbreviations

MC3165: 2-(2-((7-nitrobenzo[c][1,2,5]oxadiazol-4-yl)thio)ethoxy)ethan-1-ol
MC3181: 2-(2-(2-((7-nitrobenzo[c][1,2,5]oxadiazol-4-yl)thio)ethoxy)ethoxy)ethanol
NBDHEX: 6-((7-nitrobenzo[c][1,2,5]oxadiazol-4-yl)thio)hexan-1-ol
GST: glutathione transferase
TMZ: temozolomide
